# RNA Sequencing Reveals Differential Gene Expression of *Cerrena Unicolor* in Response to Variable Lighting Conditions

**DOI:** 10.3390/ijms20020290

**Published:** 2019-01-12

**Authors:** Anna Pawlik, Andrzej Mazur, Jerzy Wielbo, Piotr Koper, Kamil Żebracki, Agnieszka Kubik-Komar, Grzegorz Janusz

**Affiliations:** 1 Department of Biochemistry, Maria Curie-Skłodowska University, Akademicka 19 St., 20-033 Lublin, Poland; 2 Department of Genetics and Microbiology, Maria Curie-Skłodowska University, Akademicka 19 St., 20-033 Lublin, Poland; andrzej.mazur@poczta.umcs.lublin.pl (A.M.); jerzy.wielbo@poczta.umcs.lublin.pl (J.W.); piotr.koper@poczta.umcs.lublin.pl (P.K.); kamilzebracki@umcs.pl (K.Ż.); 3 Chair of Applied Mathematics and Informatics, Lublin University of Life Sciences, Akademicka 13 St., 20-950 Lublin, Poland; agnieszka.kubik@up.lublin.pl

**Keywords:** *Cerrena unicolor*, white rot fungi, light, transcriptome, RNA-seq

## Abstract

To elucidate the light-dependent gene expression in *Cerrena unicolor* FCL139, the transcriptomes of the fungus growing in white, blue, green, and red lighting conditions and darkness were analysed. Among 10,413 all-unigenes detected in *C. unicolor*, 7762 were found to be expressed in all tested conditions. Transcripts encoding putative fungal photoreceptors in the *C. unicolor* transcriptome were identified. The number of transcripts uniquely produced by fungus ranged from 20 during its growth in darkness to 112 in the green lighting conditions. We identified numerous genes whose expression differed substantially between the darkness (control) and each of the light variants tested, with the greatest number of differentially expressed genes (DEGs) (454 up- and 457 down-regulated) observed for the white lighting conditions. The DEGs comprised those involved in primary carbohydrate metabolism, amino acid metabolism, autophagy, nucleotide repair systems, signalling pathways, and carotenoid metabolism as defined using Kyoto Encyclopedia of Genes and Genomes (KEGG) database. The analysis of the expression profile of genes coding for lignocellulose-degrading enzymes suggests that the wood-degradation properties of *C. unicolor* may be independent of the lighting conditions and may result from the overall stimulation of fungal metabolism by daylight.

## 1. Introduction

Wood-colonising fungi are known to have unique biochemical pathways enabling them to assimilate a vast array of simple and complex nutrients and to produce a variety of metabolites. Most wood decay fungi are strictly saprotrophic, utilising dead wood as a food base. However, there are commonly known parasitic species of fungi (pathogens) growing and producing sporophores on living trees [1]. Elucidation of the mechanism of biological wood decay is not only important ecologically due to the role in the carbon and nitrogen cycle, but also has economic significance and plays a beneficial role in human life [2]. The environmental impact of saprotrophic fungi arises from their metabolic versatility, which includes the production of a wide range of enzymes directly or indirectly involved in the degradation of organic residues [3]. Individual wood-decomposing fungus produces various enzymes, degrades different plant material, and colonises diverse ecological niches. Therefore, understanding the physiology of wood-degrading fungi and the ability to affect their metabolic potential may have far-reaching impact for not only future research directions but also human life.

In nature, efficient lignocellulose degradation, which occurs during the process of wood decay, is mainly achieved by the activity of white rot basidiomycetes. As known, environmental factors greatly influence decay caused by white rot fungi [4], and light is one of the most important signals for every living cell. Sunlight serves as either a source of energy or information from the environment and can be considered crucial for successful competition, survival, and development in nature [5]. In fungi, light controls developmental decisions, stress response, and physiological adaptations as well as the circadian clock. To sense light, only a few photoreceptor systems have developed during evolution, comprising flavin-based blue-light, retinal-based green-light (such as rhodopsin), and linear tetrapyrrole-based red-light sensors [6]. All these major classes of photoreceptors are found in fungi suggesting that they can detect specific wavelengths by discrete receptor proteins [7]. 

The emergence of high throughput next-generation sequencing (NGS) techniques has accelerated the discovery of novel genes and has provided an opportunity to further explore and understand the differential gene expression at the genomic and transcriptional level. Since the first published transcriptome study [8], an increasing number of transcriptomes of wood decay basidiomycetes have been reported [9,10,11,12,13,14]. In contrast to the static genome, the transcriptome reflects the differential gene expression in response to developmental or environmental factors, which constitute a dynamic link between an organism’s genome and its physical characteristics [15,16,17]. Transcriptome analysis performed recently for *Cerrena unicolor*, a wood-degrading basidiomycete with great importance in biotechnology and medicine [18,19,20], revealed differential expression of genes coding for proteins engaged in degradation of various kinds of wood [14]. Moreover, the dependence of *C. unicolor* cultivation in different lighting conditions and synthesis of certain enzymes has also been demonstrated [21].

Here, we report a comparative transcriptome analysis based on high throughput RNA sequencing experiment of *C. unicolor* FCL139 cultivated in different lighting conditions. Our results provide a comprehensive dataset for analysis of transcriptional profile changes occurring in fungal cells in response to variable lighting conditions, contributing to better understanding of *C. unicolor* photobiology and light dependent metabolism and behaviour of this environmentally and commercially important fungus.

## 2. Results

### 2.1. Transcriptome Sequencing and Identification of Transcripts Coding for Fungal Photoreceptors

**Figure 1:**
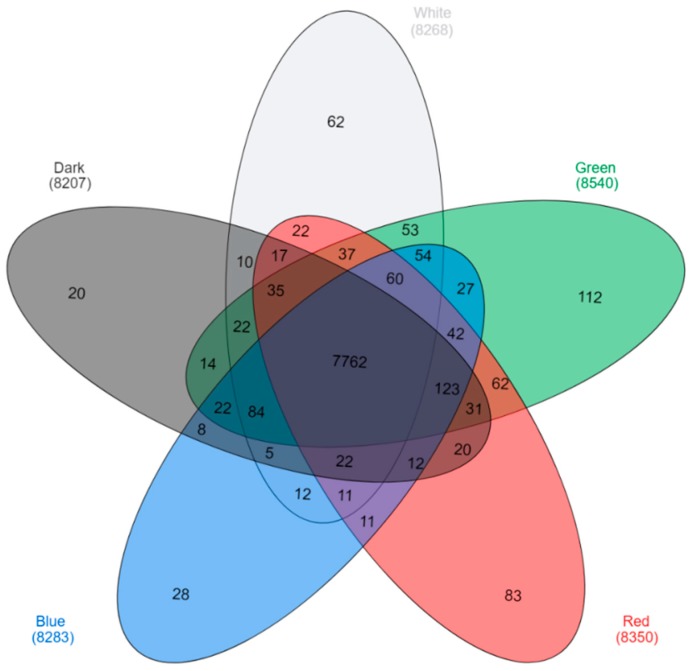
Venn diagram [22] demonstrating the number of expressed genes obtained for the tested samples. Transcripts found uniquely during *C. unicolor* growth in white light (light grey), green light (green), red light (red), blue light (blue), and dark (dark grey) are marked. The numbers of transcripts common for two, three, four, and all five tested growth conditions are indicated.

An mRNA-Seq approach was employed to gain insight into the gene expression profile of *C. unicolor* FCL139 during growth on ash sawdust in various lighting conditions comprising white (W), green (G), blue (B) and red (R) light and darkness (D) as a control. Fifteen cDNA samples (three replicates per lighting condition) were prepared from *C. unicolor* mycelia and sequenced as pair-end reads using the Solid 5500 platform resulting in a total average of 113.6 million (for *C. unicolor* cDNA samples obtained after growth in green (G) light) to 128.3 million reads (red (R) light) (Table 1). After filtering off primer-adaptor sequences, low-quality and ambiguous reads, and rRNA mapping reads, the total average number of high-quality reads ranged from 64.5 million for (G) to 93.3 million for the control (darkness, D). These high-quality filtered reads were mapped to the 12,966 predicted genes of *C. unicolor* using a Subjunc algorithm. Reads from individual sample replicates were concatenated so that there was one mapping per sample (e.g., one for white (W) light, one for green (G) light, etc.). The total number of reads that were uniquely mapped for individual samples ranged from 34.2 million for (G) to over 47.8 million for (W) (Table 1). Altogether, 10,413 all-unigenes were found for *C. unicolor* mycelia grown in various lighting conditions, among which 7762 were expressed in all tested culture variants (Figure 1).

Among *C. unicolor* all-unigenes for which a molecular function was assigned (based on the similarity of the predicted protein sequences to proteins characterised in other organisms or the presence of conserved functional domains within the predicted protein sequence), several genes encoding putative fungal photoreceptors were found (Table 2). Four of them were annotated as white collar proteins sharing similarity with respective proteins of *Neurospora crassa*. Two genes encoded proteins similar to the cryptochrome of *N. crassa,* while putative protein products of two other genes were similar to the phytochrome of *Ceratodon purpureus* and one to *Trametes versicolor* opsin (Table 2).

### 2.2. Analysis of Differentially Expressed Genes (DEGs)

**Figure A1:**
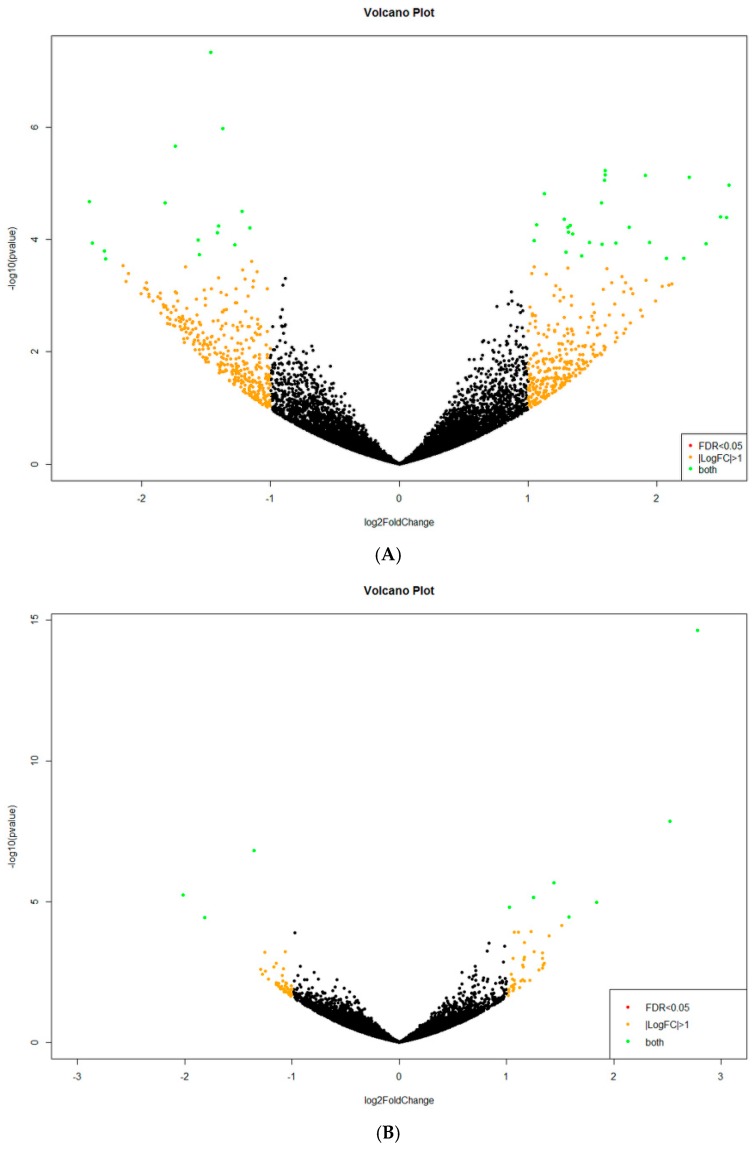

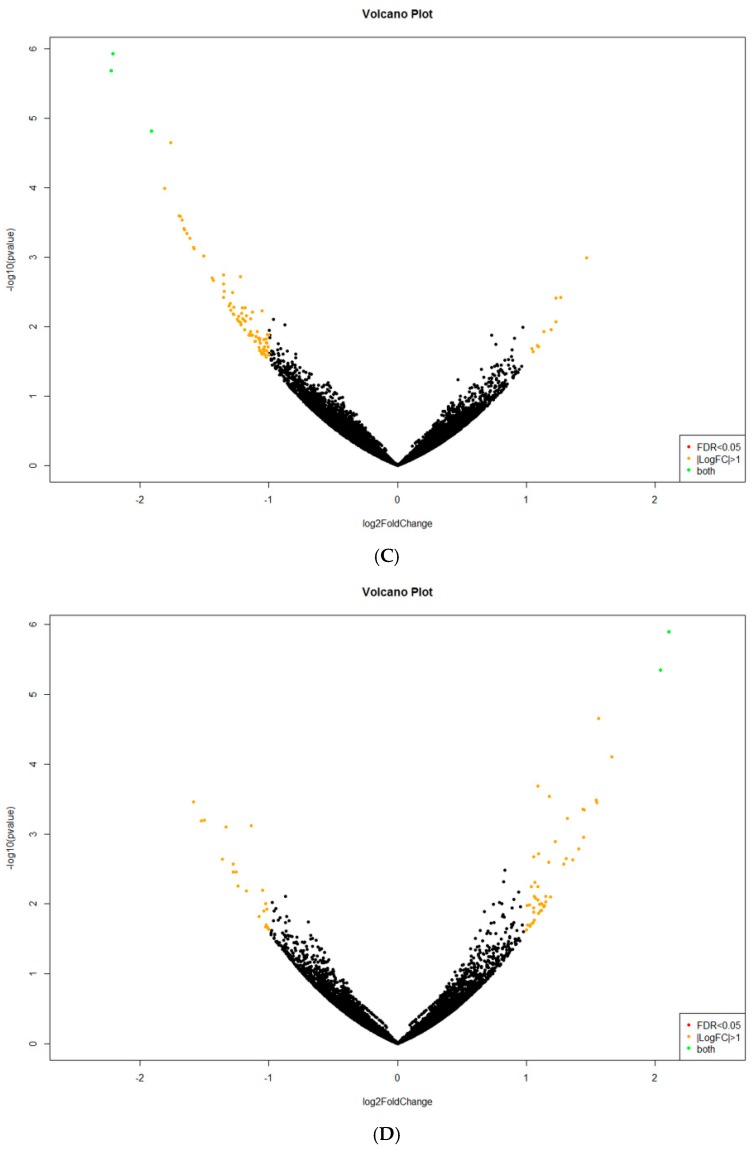
Volcano plots demonstrating *C. unicolor* genes displaying a significantly changed expression profile (large fold change values) and high statistical significance (as evidenced by the *p*-values) during fungus growth in different lighting conditions and darkness: (**A**) white vs. dark; (**B**) blue vs. dark; (**C**) green vs. dark; and (**D**) red vs. dark. The data for all detected transcripts are plotted as log2 fold change (X-axis) versus the −log10 of the adjusted *p*-value (Y-axis). Significantly up-regulated or down-regulated genes (at least four-fold at *p* < 0.05) are highlighted as green dots.

For the analysis of DEGs, genes that were expressed with fragments per kilo base per million reads (FPKM) >1 in at least one of the lighting variants were included and considered as “expressed genes”. The number of “expressed genes” varied between the tested lighting variants and was 8267 for W, 8539 for G, 8282 for B, 8349 for R, and 8206 for D. The analysis of *C. unicolor* transcriptomes revealed numerous genes whose expression differed significantly (*p*-value <0.05) between the dark variant (control) and each of the light variants used (Table 3 and Figure A1). In general, the greatest number of DEGs (454 up-regulated and 457 down-regulated) was observed when the fungus was grown in the white light, compared to the control conditions. In contrast, the lowest number of DEGs (45 up-regulated and 171 down-regulated) was observed during *C. unicolor* growth in the green light, compared to the control. Only in the white lighting variant, the numbers of up-regulated and down-regulated genes were similar. In the other tested conditions (except for red vs. darkness), down-regulated genes predominated (Table 3). 

### 2.3. KEGG Pathway Enrichment of DEGs

**Figure 2:**
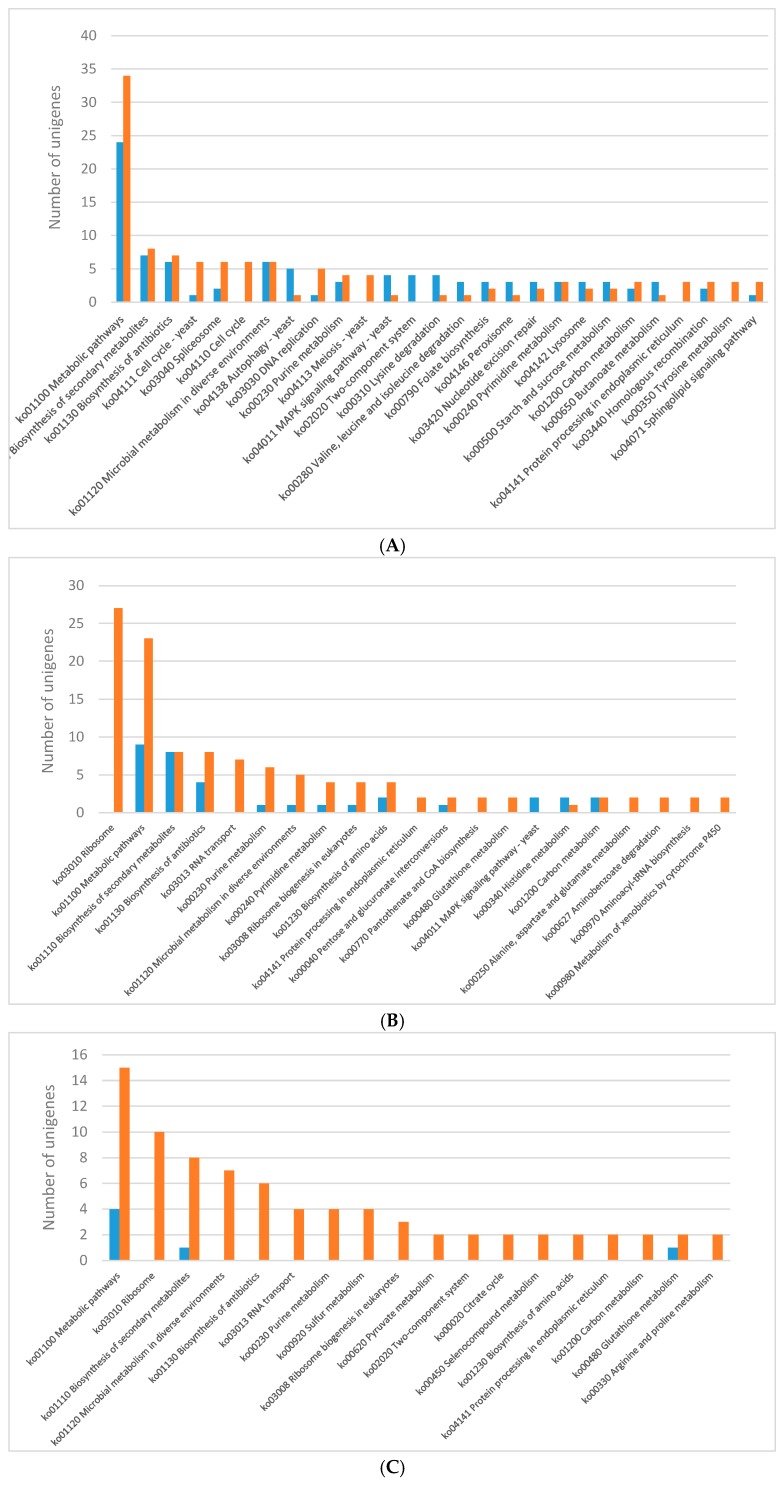

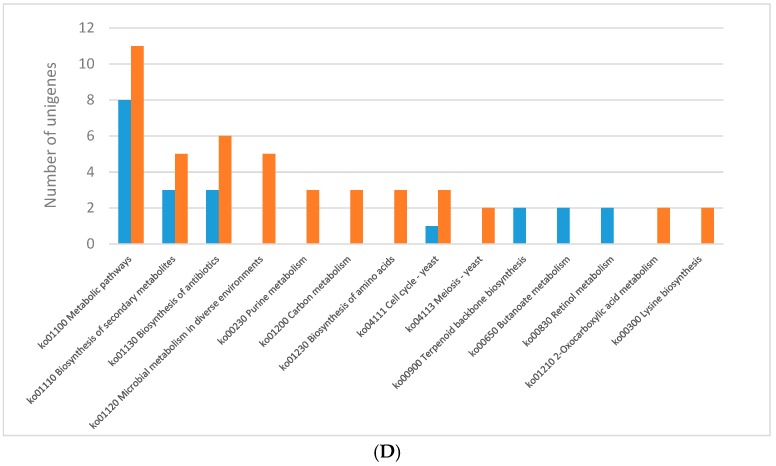
Results of the KEGG pathway analysis of differentially expressed genes (DEGs) obtained for the following *C. unicolor* light culturing conditions: (**A**) white vs. dark; (**B**) blue vs. dark; (**C**) green vs. dark; and (**D**) red vs. dark. The number of specific up-regulated (blue bar) and down-regulated (red bar) unigenes is indicated.

The KEGG pathway analysis was performed for differentially expressed genes (Figure 2). Generally, most of the DEGs were ascribed into the “metabolic pathways” category in almost all lighting conditions except for the blue light. The genes encoding various metabolic pathways were mostly down-regulated in each of the analysed lighting variants (Figure 2). The genes representing KEGG pathways related to biosynthesis of secondary metabolites and antibiotics were fairy abundant in all the lighting variants as well. The blue light specifically induced expression of genes potentially engaged in histidine metabolism, while the expression of genes related to retinol metabolism was up-regulated in mycelia grown in the red light. Specific induction of the genes encoding putative proteins belonging to a two-component system, MAPK and RIG-I-like receptor-based signalling pathways, lysine degradation, sphingolipid and pyruvate metabolism, DNA repair pathways, endocytosis and autophagy was observed in the white light conditions. Only a few KEGG categories were found as unique in individual lightning variants when down-regulated genes were considered in the pathway analysis. In the mycelia grown in the blue and green light, a number of down-regulated genes related to ribosome biogenesis were observed. Fungus growth in the red light resulted in repression of putative genes engaged in lysine synthesis, while the green light specifically down-regulated genes involved in the citric cycle. The expression of genes related to the cell cycle, DNA replication, and tyrosine metabolism pathways was found to be down-regulated in *C. unicolor* grown in the white light, whereas all genes whose expression was influenced by the blue light fell into categories shared by at least one other light variant (Figure 2). 

#### 2.3.1. Light Regulates Primary Metabolism of *C. Unicolor*

The KEGG pathway analysis revealed that light exerted a global influence on the primary metabolism of *C. unicolor*. In comparison to darkness (control), during fungus growth in the white lighting conditions, substantially decreased expression of three genes encoding putative enzymes, namely methylenetetrahydrofolate reductase (NADPH) [EC 1.5.1.20], gluconokinase [EC 2.7.1.12], and acetyl-CoA synthetase [EC 6.2.1.1] was observed, which may further affect carbon metabolism. On the other hand, up-regulation of genes encoding putative D-lactate dehydrogenase (cytochrome) [EC 1.1.2.4] and acetyl-CoA C-acetyltransferase [EC 2.3.1.9] may enhance pyruvate metabolism. Moreover, down-regulation of acylpyruvate hydrolase [EC 3.7.1.5], 4-hydroxy-2-oxoheptanedioate aldolase [EC 4.1.2.52], and tyrosinase [EC 1.14.18.1] encoding genes was observed during fungus growth in the white light culture variant. The genes were potentially engaged in the metabolism of tyrosine leading to its transformation to pyruvate, succinate, or melanin. Most of the genes whose expression was repressed during *C. unicolor* growth in the white light encoded putative enzymes are involved in purine/pyrimidine metabolism, DNA replication, and homologous recombination or participating in protein processing in the endoplasmic reticulum. On the other hand, the white light induced expression of putative enzymes that degrade lysine, isoleucine, leucine, and valine and enhance degradation of fatty acids. Starch and sucrose metabolism seemed to be influenced in the mycelia growing in the white lighting conditions by the up-regulated expression of genes coding for trehalose 6-phosphate synthase [EC 2.4.1.15 2.4.1.347], β-glucosidase [EC 3.2.1.21], and trehalose 6-phosphate synthase/phosphatase [EC 2.4.1.15 3.1.3.12]. The cultivation of *C. unicolor* in the blue lightning conditions induced expression of putative enzymes involved in histidine metabolism (glutamine amidotransferase/cyclase [EC 2.4.2.- 4.1.3.-], l-histidine *N*-α-methyltransferase/hercynylcysteine S-oxide synthase [EC 2.1.1.44 1.14.99.51]). Concomitantly, the expression of genes encoding proteins of large and small ribosomal subunits and other genes that may be involved in protein synthesis (translation factors eIF-2B; 3, 4E), amino acid synthesis (d-3-phosphoglycerate dehydrogenase/2-oxoglutarate reductase [EC 1.1.1.95 1.1.1.399], saccharopine dehydrogenase (NADP+, l-glutamate forming) [EC 1.5.1.10], glutamine amidotransferase/cyclase [EC 2.4.2.- 4.1.3.-], and argininosuccinate synthase [EC 6.3.4.5]) and in protein processing in ER (ubiquitin-conjugating enzyme E2 G1 [EC 2.3.2.23] and translocation protein SEC62) was down-regulated by the blue light. 

Similar to the blue light, the green lighting variant of fungus growth resulted in decreased expression of genes coding for large and small ribosomal subunit proteins, translation initiation factor 3 subunit C, and arginine/proline metabolism (glutamate-5-semialdehyde dehydrogenase [EC 1.2.1.41] and agmatinase [EC 3.5.3.11]). Moreover, purine metabolism seemed to be affected by down-regulation of genes coding for sulphate adenylyltransferase [EC 2.7.7.4], DNA-directed RNA polymerases I, II, and III subunit RPABC5, phosphoribosylaminoimidazole carboxylase [EC 4.1.1.21], and adenylate kinase [EC 2.7.4.3]. The expression of the primary pathway enzymes, presumably belonging to the pyruvate metabolism and citric cycle (malate dehydrogenase [EC 1.1.1.37] and pyruvate dehydrogenase E1 component alpha subunit [EC 1.2.4.1]), was also down-regulated by the green light. No putative enzymes involved in primary metabolism were specifically induced during fungus growth in the red light. However, this lighting variant decreased the expression of genes coding for enzymes engaged in purine metabolism (ATP adenylyltransferase [EC 2.7.7.53], AMP deaminase [EC 3.5.4.6], and DNA-directed RNA polymerase III subunit RPC2 [EC 2.7.7.6]), carbon metabolism (glycine dehydrogenase [EC 1.4.4.2], methylenetetrahydrofolate reductase (NADPH) [EC 1.5.1.20], and 6-phosphogluconolactonase [EC 3.1.1.31]) and amino acid biosynthesis (homoaconitate hydratase [EC 4.2.1.36], argininosuccinate synthase [EC 6.3.4.5], and homoaconitase [EC 4.2.1.-]).

#### 2.3.2. Effect of Light on the Expression of Wood-Degrading Enzymes

Since *C. unicolor* is a white rot fungus and the mycelia were cultivated on ash sawdust during this experiment, analysis of the expression pattern of genes coding for wood degrading enzymes was conducted. Transcripts of genes encoding all groups of enzymes (cellulases, hemicellulases, and lignin-modifying enzymes) were found in the transcriptomes of *C. unicolor* cultivated in the tested lightning conditions (Table 4). However, the highest number of transcripts of genes related to wood degradation (16, comprising both up- or down-regulated genes) was noted when white light was applied during fungus growth, while the lowest number (only four transcripts) was recorded in the green lighting variant. In all lightning conditions except for the green light variant, the up-regulated genes related to wood degradation predominated. The white light induced mainly the expression of genes encoding lignin-degrading auxiliary (LDA) enzymes comprising alcohol oxidase (XLOC_000665) and five cytochromes P-450 (XLOC_007791, XLOC_005202, XLOC_006061, XLOC_007410, and XLOC_001403). Similarly, the blue light induced the expression of putative LDA enzymes including aryl-alcohol dehydrogenase (XLOC_008717), pyranose 2-oxidase (XLOC_009983), and monooxygenase (XLOC_003435) and the red light influenced the expression of cytochrome P-450 (XLOC_007791, XLOC_00671, XLOC_006683, XLOC_001969, and XLOC_007717). On the other hand, the fungus growth in the red light variant resulted in down-regulation of the laccase coding gene (XLOC_011551), whereas the white light repressed four laccase genes (XLOC_008979, XLOC_008955, XLOC_000669, and XLOC_011551). The expression of genes coding for all hemicellulases and almost all cellulases was induced by the different lightning conditions. Only exoglucanase (XLOC_006587) and endo-β-1,4-glucanase (XLOC_007951) coding genes were down-regulated when the blue and white light variants were applied, respectively.

### 2.4. Signalling Pathways

**Figure 3:**
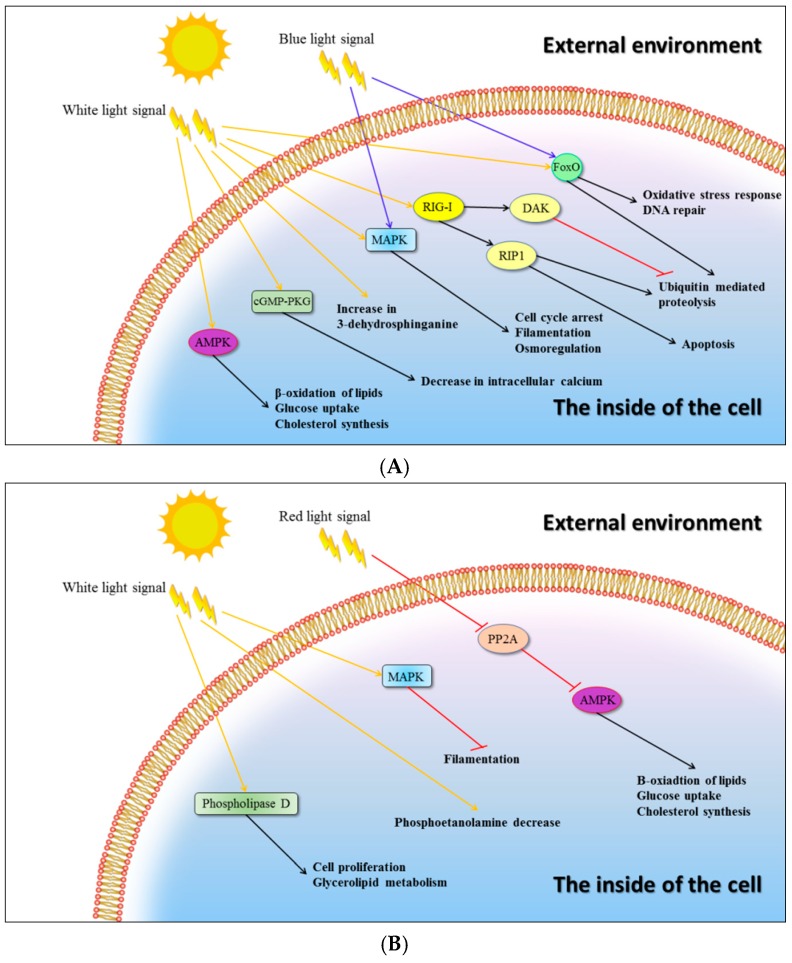
Putative light-regulated signalling pathways in *C. unicolor* FCL139. The effect of white (W), blue (B), and red (R) light on the up-regulation (**A**) and down-regulation (**B**) of genes coding for putative signal-transducing molecules and their engagement in metabolic pathways has been indicated. Arrow-headed lines and bar-headed lines indicate activation and inhibition, respectively. The colour of an arrow-headed line corresponds to the specific wavelength of light.

Besides the above-mentioned genes coding for enzymes that are directly engaged in *C. unicolor* primary metabolism and wood degradation, the transcripts of several genes coding for proteins of various signalling pathways were detected in the mycelia cultured in the different lightning conditions, indicating that light may be a regulatory factor affecting other cellular processes (Figure 3 and Table A1). It was observed that the white and blue light induced the expression of the mitogen-activated protein kinase (MAPK) gene, which may be responsible for signal transmission to the pathways related to filamentation, osmoregulation, or cell cycle arrest. Both the white and blue light variants also induced the FoxO signalling pathway (the “Forkhead box” (FOX)). However, the blue light signal seemed to be further transduced via FoxO to the processes of oxidative stress response and DNA repair, whereas the white light may have affected ubiquitin mediated proteolysis. The latter process may also have been affected by the white light via an alternative signalling pathway involving a RIG-I-like receptor, whose expression was induced in the studied conditions. The intracellular calcium level may be additionally regulated by white light via induced expression of cGMP-dependent protein kinase (cGMP-PKG). Several primary metabolism pathways comprising β-oxidation of lipids, glucose uptake, and cholesterol synthesis may be indirectly regulated by white light affecting the AMP-activated protein kinase (AMPK) pathway. The primary metabolic processes mentioned above may also be indirectly regulated by protein phosphatase 2 (PP2A, a known inhibitor of AMPK), whose expression was negatively affected by the red light.

## 3. Discussion

Reaction of fungi to illumination is multidirectional, and light can significantly enhance their fitness [5]. Despite the knowledge about the prevalence of photoreceptors in fungi, the influence of light on metabolic processes has not been investigated in detail in most cases. The analyses of fungi response to light is predominantly restricted to white light or daylight effects vs. darkness as the most extreme values [5,7]. In this work, transcriptomic response to the different wavelengths of light was studied in *C. unicolor* FCL139. From 20 to 112 transcripts were found to be specific to one of the culturing conditions, suggesting that *C. unicolor* can respond to specific light wavelengths in different manner. This observation is in agreement with the previous studies and the finding that even significant number of transcribed genes of organisms may be engaged in response to light [5,23]. In *N. crassa,* 31% of the expressed genes were affected two-fold or more by exposure to light [24]. Among all assigned unigenes of *C. unicolor*, those coding for putative photoreceptor proteins were found. The presence of transcripts coding for putative proteins similar to *N. crassa* white collar proteins and cryptochromes, *C. purpureus* phytochromes, and *T. versicolor* opsins suggests that *C. unicolor* is able to sense from blue to far-red light. The similarity of *C. unicolor* receptors to plant phytochromes may be surprising. It should be noticed that no phytochrome-like light sensor protein has been characterised in basidiomycetes, and plants and fungi share the same structure of the conserved photosensing region of the receptor [25], which may suggest an unusual structure of the carboxy-terminal domain in *C. unicolor*. 

The analysis of DEGs performed for *C. unicolor* revealed numerous genes whose expression varies considerably during fungus growth. The KEGG pathway analysis of differentially expressed genes revealed significant enrichment of metabolic pathways indicating that light influenced mainly primary metabolism of *C. unicolor*. The expression of genes engaged in metabolic pathway was mostly down-regulated in each of the analysed lighting variant except for blue light. Concomitantly, in white light conditions, up-regulation of genes engaged in amino acid degradation, autophagy, nucleotide repair systems, signalling pathways, and carotenoid metabolism was observed. In turn, 319 differentially expressed carbohydrate-active enzymes participated in carbon metabolism were identified in *Pleurotus eryngii* under blue light stimulation [26]. In *N. crassa*, light increased cellular metabolism causing simultaneously significant oxidative stress to the organism. In response to this stress, protective photopigments and antioxidants were produced, and genes involved in ribosome biogenesis were transiently repressed [24]. The number of down-regulated genes related to ribosome biogenesis was also observed in the *C. unicolor* mycelia grown in the blue and green light. In *Aspergillus ornatus*, glucose metabolism was decreased by light prior to production of conidia [5]. While blue light is a commonly known signal triggering up-regulation of carotenogenesis [27], the red light induction of genes related to respective pathway was observed in *C. unicolor*. Transcriptome analysis of *Cordyceps militaris* albino mutant and its sibling strain revealed that the previously identified photoreceptors were expressed similarly in response to light. Both strains shared 235 DEGs after the light treatment and all DEGs were grouped into three functional categories: biological processes, cellular components, and molecular functions [28]. Similar discrepancies in the metabolism in different fungal species were previously reported in two model organisms: *N. crassa* and *Aspergillus nidulans* [29]. Light triggers significant expressional changes within the first 24–48 h in *A. nidulans* and darkness results in a massive transcriptional reprogramming of gene expression [30].

In *Trichoderma reesei* cultivated in darkness, significant increase in transcripts coding for cellulases was observed [31]. This phenomenon may be regulated by cAMP-dependent protein kinase (PKA), which influences cellulase synthesis positively in light and negatively in dark conditions. In *C. unicolor* positive changes in the number of transcripts involved in the AMPK pathway was observed in white lighting. However, only one transcript coding for cellulase was found to be up-regulated in these conditions. Tang et al. [32] proved that light-induced brown film formation in *Lentinula edodes* occurred via MAPK and a two-component system, with possible engagement of white collar proteins or phytochromes. Our results show that both pathways (AMPK and MAPK based) were induced by the white light in *C. unicolor*. Moreover, up-regulation of genes coding for cytochrome P-450 and monooxygenase by the red and blue light, respectively, which was observed in our analysis, seem to support the role of putative white collar proteins or phytochromes in AMPK and MAPK based signal transduction. Phytochromes as red light receptors were also reported to be involved in regulation of secondary metabolism in *A. nidulans* [33] and our data suggest that synthesis of several enzymes involved in secondary metabolism was induced by the light of same wavelength. Furthermore, in *C. unicolor*, the MAPK pathway induction by the white light resulted in enhanced filamentation, which was proved to be a stress response in bacteria to DNA damage [34]. MAPK was suggested to play an important role as part of the DNA repair system in *Trichoderma atroviride* [35]. In turn, PP2A was proved to be part of the FREQUENCY(FRQ)-based oscillator with circadian properties in *N. crassa* [36]. PP2A expression was down-regulated by red light in our experiment, raising the possibility that *C. unicolor* is sensitive to day-night changes. 

Recently reported comparative transcriptomic analysis of *C. unicolor* performed in uncontrolled lighting conditions revealed differential expression of genes engaged in wood degradation during fungus growth on ash, maple, and birch sawdust, and significant differences were observed for cellulase-coding genes [14]. Currently, transcripts of genes coding for cellulases, hemicellulases, and lignin-modifying enzymes were found during fungus growth on ash sawdust medium in all lighting conditions, which may suggest that wood-degradation properties of *C. unicolor* depend on the medium applied rather than lighting conditions [14,37]. The high number of transcripts related to wood degradation observed during fungus growth in the white light may have resulted from the overall stimulation of its metabolism by daylight [38]. It has been demonstrated recently that blue light efficiently boosted laccase synthesis in *C. unicolor* and *Pycnoporus sanguineus*, whereas in *Phlebia lindtneri* the highest laccase activity were observed in green lighting conditions in LH or cellulose-based medium [21]. Laccase was also up-regulated 10 h after blue light exposure in *Coprinopsis cinerea* [39]. On the contrary, green light efficiently inhibited laccase in other *P. sanguineus* strains and maximum enzyme activity was noticed in the darkness [40]. 

In conclusion, understanding how *C. unicolor* and other fungi sense the environment is of intellectual, industrial, and medicinal importance. The detection of transcripts encoding for putative fungal photoreceptors in *C. unicolor* transcriptome strongly suggests that the fungus can sense light and respond to various wavelength of light in a slightly diverse manner, by changing its overall metabolism. The up-regulation of gene expression of several signalling pathways, which can be considered as a hub integrating and transducing various environmental signals, implies that light together with other factors may affect cellular processes of the fungus.

## 4. Materials and Methods 

### 4.1. Medium and Growth Conditions

The *C. unicolor* FCL139 strain was obtained from the culture collection of the Regensberg University. The fungal culture was maintained in 2% (*w*/*v*) malt agar slants. As an inoculum, pieces of agar culture were grown in the Lindeberg and Holm (LH) medium [41] in non-agitated conical flasks for 10 days at 28 °C. Next, the mycelial mats were collected and homogenised in a disperser (IKA, Warszawa, Poland). The fragmented mycelial culture (1% *v*/*v*) was used as a standard inoculum for further studies. Solid-state lignocellulose *C. unicolor* cultures were grown at 28 °C on 1 g of sterile ash sawdust (wood particles < 4 mm) soaked with 9 mL of distilled water and solidified with 1.5 % of agar in Petri dishes covered with a nylon membrane filter (0.45 μm). The seeds were then cultivated at 28 °C in incubators equipped with illumination led cassettes (KT 115, Binder, Neckarsulm, Germany) for 8 days. Continuous lighting conditions (20 lux) were provided throughout the entire period of *C. unicolor* cultivation. The following lighting variants were applied: white (W) (4000–4750 K), green (G) (510–520 nm), blue (B) (465–470 nm), and red (R) (620–625 nm) light and darkness (D) as a control.

### 4.2. RNA Extraction and Sequencing

Fifty milligrams of *C. unicolor* mycelia were frozen in liquid nitrogen and hand ground to a fine powder with a pre-chilled mortar and pestle. Polyadenylated (polyA) mRNA was directly isolated using oligo (dT) magnetic beads. The Dynabeads mRNA DIRECT Kit (Ambion, Waltham, MA, USA) designed for simple and rapid isolation of polyA mRNA directly from the crude lysate was employed for polyA mRNA extraction following the manufacturer’s protocol. After homogenisation of hyphae, the DNA shearing step (allowing reduction of viscosity) was performed by forcing the lysate 3–5 times through a 21-gauge needle using a 2 mL syringe. The mRNA quantity and quality were evaluated using a Qubit 2.0 fluorimeter and a Qubit RNA HS Assay Kit (Thermo Fisher Scientific, Waltham, MA, USA), as well as an Agilent 2100 Bioanalyzer and an RNA 6000 Nano Kit (Agilent Technologies, Santa Clara, CA, USA). The transcriptome library for each sample was prepared using a Solid Total RNA-Seq Kit (Applied Biosystems, Waltham, MA, USA) following the manufacturer protocol for low-input RNA samples. The median size of the mRNA fragments after RNase III fragmentation ranged from 138 to 195 bp. The median size of the amplified cDNA whole transcriptome libraries ranged from 225 to 356 bp. Each of the 15 constructed cDNA libraries (3 replicates per lighting variant) was sequenced using the Solid 5500 platform. The sequencing data were derived from the paired-end reads (50 bp forward and 35 bp reverse) of the template in the Solid templated bead, using forward and reverse ligation chemistry (F3, FWD1 and F5, RNA REV1 Seq. Primers, respectively) in a single run. The processing of original images to sequences and base-calling for the obtained paired-end reads were performed by the LifeScope pipeline (version 1.6) (Life Technologies, Carlsbad, CA, USA).

### 4.3. NGS Data Analysis

The SOLiD sequencing results of paired-end reads in triplicates for each *C. unicolor* sample were converted into FASTQ format using the solid2fastq program from the BFAST package [42] with default settings. Adapter trimming and removal of low-quality reads was performed with cutadapt [43]. Filtered and processed results of individual cDNA replicates reads of *C. unicolor* are summarised in Table 1. The *C. unicolor* rRNAs were identified de novo with Barrnap 0.7 (Available online: https://github.com/Victorian-Bioinformatics-Consortium/barrnap). Based on these predictions, rRNA sequences were fetched from the *C. unicolor* genome using a custom Python script, and the rRNA-mapping reads were excluded from the sequencing results using Bowtie 2 [44]. The *C. unicolor* v1.1 (Cerun2) genome assembly, downloaded from the U.S. Department of Energy Joint Genome Institute (DOE JGI, http://jgi.doe.gov), was used as the reference for the mapping of the obtained filtered reads. Reads from each of the sample replicates were concatenated using a Subjunc algorithm of the Subread software package so that there was one mapping per sample to the reference genome [45]. Re-annotation of the *C. unicolor* genome based on available JGI annotation and high-throughput sequence data of five transcriptomes was performed with the Cufflinks software package [46]. The gene and transcript identifiers assigned by Cufflinks were used in further analyses. The default BLAST E-value cutoff 10^−5^ was used during similarity searches.

Transcript abundance was calculated on the basis of the number of mRNA-Seq reads mapped to a given gene model with RSEM, accurate transcript quantification software, from RNA-Seq data [47] and normalised to TPM (transcripts per million) and FPKM (fragments per kilobase of exon per million fragments mapped) values, respectively. Identification of the differentially expressed genes (DEGs) based on the abundance of transcripts and isoforms of the same gene between mycelia grown in darkness (control) and each of the lighting variants was performed with the DESeq 2 package [48].

Individual genes and transcripts were automatically annotated on the basis of predicted sequence similarities to known proteins of the SwissProt and TrEMBL databases as well as the presence of conserved domains (PFAM database).

### 4.4. Nucleotide Sequence Accession Numbers

The raw data from RNAseq experiments have been deposited to the Sequence Read Archive database of NCBI under BioProject number PRJNA401095.

## Figures and Tables

**Table 1:** Filtering and processing of individual cDNA replicate sample reads of *C. unicolor* grown on ash sawdust in the following lighting variants: white (W), green (G), blue (B), and red (R) light and darkness (D) as a control.

Sample Name	Number of Total Reads	Average Number of Reads per Lighting Condition	Per cent of too Short Reads	Per cent of Reads with too Many Ns	Number of rRNA Mapped Reads	Number of Reads which Passed Filtering, Quality Control, rRNA Removal (% of Total Reads) ^1^	Number of Mapped Reads ^1^
W_1	112,287,853	120,864,091	16.4%	69.4%	458,752	15,517,301 (13.82%)	47,884,777
W_2	121,609,141		22.3%	1.9%	1,815,166	90,460,336 (74.39%)	
W_3	128,695,279		13.9%	1.2%	3,393,460	105,877,886 (82.27%)	
G_1	128,090,596	113,654,401	17.0%	59.7%	437,929	29,427,455 (22.97%)	34,288,490
G_2	131,301,239		19.2%	2.2%	1,151,865	102,046,831 (77.72%)	
G_3	81,571,367		20.3%	1.0%	2,134,477	62,027,269 (76.04%)	
B_1	120,729,656	128,318,401	17.8%	1.0%	2,226,961	95,742,071 (79.30%)	47,208,269
B_2	121,879,416		20.6%	2.1%	1,342,182	92,929,241 (76.25%)	
B_2	142,346,130		36.5%	0.9%	1,787,916	87,349,092 (61.36%)	
R_1	141,730,254	128,365,999	16.2%	58.6%	591,388	35,131,581 (24.79%)	40,335,011
R_2	121,611,135		22.3%	1.9%	1,519,062	90,665,568 (74.55%)	
R_3	121,756,608		19.8%	1.0%	2,341,637	94,055,653 (77.25%)	
D_1	139,936,639	123,716,947	13.9%	1.1%	3,078,557	115,934,413 (82.85%)	48,645,244
D_2	134,402,468		23.0%	1.7%	989,101	100,168,832 (74.53%)	
D_3	96,811,733		31.3%	1.8%	918,966	63,824,504 (65.93%)	

^1^ Reads from each sample replicates were subsequently concatenated so at the stage of mapping to the reference genome there was just one mapping per lighting conditions.

**Table 2:** Transcripts encoding putative photoreceptors identified in *C. unicolor* transcriptome during fungus growth in the different lighting conditions.

Gene ID	SwissProt Best Hit	Putative Function
XLOC_005534	SwissProt_best_hit “White collar 2 protein (7e-12, sp|P78714|WC2_NEUCR)”	white collar protein
XLOC_005717	SwissProt_best_hit “White collar 2 protein (1e-11, sp|P78714|WC2_NEUCR)”	white collar protein
XLOC_007605	SwissProt_best_hit “White collar 1 protein (8e-63, sp|Q01371|WC1_NEUCR)”	white collar protein
XLOC_011520	SwissProt_best_hit “White collar 1 protein (1e-10, sp|Q01371|WC1_NEUCR)”	white collar protein
XLOC_008457	SwissProt_best_hit “Deoxyribodipyrimidine photo-lyase (6e-97, sp|P27526|PHR_NEUCR)”	cryptochrome
XLOC_003467	SwissProt_best_hit “Deoxyribodipyrimidine photo-lyase (6e-97, sp|P27526|PHR_NEUCR”	cryptochrome
XLOC_010394	SwissProt_best_hit “Light-sensor Protein kinase (2e-08, sp|P25848|PHY1_CERPU”	phytochrome
XLOC_011661	Light-sensor Protein kinase (4e-17, sp|P25848|PHY1_CERPU)	phytochrome
XLOC_003467	Protein FDD123 (1e-30, sp|O74631|FD123_TRAVE)	opsin

**Table 3:** Number of *C. unicolor* genes whose expression differed significantly (at *p* < 0.05), during fungus growth in the tested lighting conditions in comparison to the control (darkness). Only genes whose transcripts abundance (FPKM) in at least one of the tested conditions was >1 were considered.

Compared Lightning Conditions	Number of Differentially Expressed Genes (at *p* < 0.05)	
	Up-Regulated	Down-Regulated
blue vs. dark	176	227
green vs. dark	41	167
red vs. dark	132	95
white vs. dark	454	457

**Table 4:** *C. unicolor* differentially expressed genes encoding for wood-degrading enzymes detected during fungus growth in different lighting conditions.

Gene ID	Blast Best Hit	Putative Function	Expression	
			log2 Fold Change	*p*-Value
**White vs. Dark**				
XLOC_007791	Cytochrome P450 3A11 (2e-20, sp|Q64459|CP3AB_MOUSE)	LDA ^1^	1.527678	0.012165
XLOC_005202	Cytochrome P450 52A5 (7e-52, sp|Q12581|CP52X_CANMA)	LDA ^1^	1.312698	0.03231
XLOC_004360	Manganese peroxidase 3 (2e-75, sp|Q96TS6|PEM3_PHLRA)	LME ^2^	1.310116	0.015162
XLOC_006405	Beta-glucuronidase (3e-14, sp|A2QEQ6|GUS79_ASPNC)	hemicellulase	1.18915	0.013524
XLOC_007195	Beta-glucosidase cel3A (3e-36, sp|G4NI45|CEL3A_MAGO7)	cellulase	1.186582	0.028688
XLOC_006061	Cytochrome P450 734A1 (2e-23, sp|O48786|C734A_ARATH)	LDA ^1^	1.015716	0.001687
XLOC_007410	Cytochrome P450 4A10 (3e-20, sp|P08516|CP4AA_RAT)	LDA ^1^	0.996072	0.016423
XLOC_000665	Alcohol oxidase (6e-93, sp|P04841|ALOX_PICAN)	LDA ^1^	0.953991	0.014911
XLOC_001403	Cytochrome P450 67 (Fragment) (4e-60, sp|O00061|CP67_UROFA)	LDA ^1^	0.936259	0.012773
XLOC_007076	Xyloglucanase (0.0, sp|Q7Z9M8|XG74_HYPJQ)	hemicellulase	0.863734	0.04043
XLOC_002989	Endo-1,4-beta-xylanase C (2e-132, sp|B7SIW2|XYNC_PHACH)	hemicellulase	0.855198	0.017668
XLOC_008979	Laccase (1e-32, sp|O59896|LAC1_PYCCI)	LME ^2^	−0.67001	0.031583
XLOC_008955	Laccase (2e-170, sp|Q02497|LAC1_TRAHI)	LME ^2^	−0.92358	0.037087
XLOC_007951	Probable endo-beta-1,4-glucanase D (3e-34, sp|B0Y9G4|EGLD_ASPFC)	cellulase	−1.01988	0.044786
XLOC_000669	Laccase (2e-80, sp|Q01679|LAC1_PHLRA)	LME ^2^	−1.172	0.045441
XLOC_011551	Laccase-2 (0.0, sp|Q12718|LAC2_TRAVE)	LME ^2^	−1.53184	0.008822
**Blue vs. Dark**				
XLOC_006977	Probable beta-glucosidase G (2e-15, sp|B8NMR5|BGLG_ASPFN)	cellulase	1.398314	0.00017
XLOC_008717	Aryl-alcohol dehydrogenase [NADP(+)] (4e-39, sp|Q01752|AAD_PHACH)	LDA ^1^	0.940399	0.030909
XLOC_009983	Pyranose 2-oxidase (3e-32, sp|Q6QWR1|P2OX_PHACH)	LDA ^1^	0.905345	0.041241
XLOC_008418	Beta-glucuronidase (2e-06, sp|A2QEQ6|GUS79_ASPNC)	hemicellulase	0.640958	0.04928
XLOC_003435	Putative monooxygenase Rv1533 (1e-10, sp|O06179|Y1533_MYCTU)	LDA ^1^	0.615906	0.043912
XLOC_001060	Cytochrome P450 67 (Fragment) (2e-43, sp|O00061|CP67_UROFA)	LDA ^1^	−0.4461	0.046049
XLOC_006587	Exoglucanase 1 (2e-111, sp|P13860|GUX1_PHACH)	cellulase	−0.47763	0.048334
**Green vs. Dark**				
XLOC_006977	Probable beta-glucosidase G (2e-15, sp|B8NMR5|BGLG_ASPFN)	cellulase	0.93716	0.044217
XLOC_006843	Xyloglucan-specific endo-beta-1,4-glucanase A (7e-61, sp|Q5BG78|XGEA_EMENI)	hemicellulase	0.736937	0.038777
XLOC_008194	Aryl-alcohol dehydrogenase [NADP(+)] (8e-126, sp|Q01752|AAD_PHACH)	LDA ^1^	−0.85863	0.037114
XLOC_010403	Cytochrome P450 3A30 (4e-16, sp|Q9PVE8|C330_FUNHE)	LDA ^1^	−1.78285	2.49 × 10^−5^
**Red vs. Dark**				
XLOC_007791	Cytochrome P450 3A11 (2e-20, sp|Q64459|CP3AB_MOUSE)	LDA ^1^	2.048597	4.47 × 10^−6^
XLOC_006731	Cytochrome P450 monooxygenase yanC (9e-49, sp|G3Y416|YANC_ASPNA)	LDA ^1^	1.552875	0.000342
XLOC_000034	Probable mannan endo-1,4-beta-mannosidase F (1e-50, sp|Q5AR04|MANF_EMENI)	hemicellulase	1.451517	0.001082
XLOC_006683	Cytochrome P450 monooxygenase yanC (2e-28, sp|G3Y416|YANC_ASPNA)	LDA ^1^	1.315805	0.002199
XLOC_002405	Aryl-alcohol dehydrogenase [NADP(+)] (3e-171, sp|Q01752|AAD_PHACH)	LDA ^1^	1.295598	0.002627
XLOC_001969	Cytochrome P450 monooxygenase yanC (3e-104, sp|G3Y416|YANC_ASPNA)	LDA ^1^	0.917329	0.031318
XLOC_010055	Versatile peroxidase VPL1 (9e-155, sp|Q9UR19|VPL1_PLEER)	LME ^2^	0.861786	0.037494
XLOC_007717	Cytochrome P450 monooxygenase yanC (0.0005, sp|G3Y416|YANC_ASPNA)	LDA ^1^	0.828494	0.04104
XLOC_004247	Manganese peroxidase 3 (6e-50, sp|Q96TS6|PEM3_PHLRA)	LME ^2^	0.819839	0.014128
XLOC_011551	Laccase-2 (0.0, sp|Q12718|LAC2_TRAVE)	LME ^2^	−1.27902	0.003542

^1^ LDA, lignin-degrading auxiliary enzyme; ^2^ LME, lignin-modifying enzyme.

## Abbreviations

KEGG	Kyoto Encyclopedia of Genes and Genomes
DEGs	Differentially Expressed Genes
LDA	Lignin-Degrading Auxiliary Enzyme
LME	Lignin-Modifying Enzyme

## Appendix B

**Table A1:** *C. unicolor* differentially expressed genes engaged in signalling pathways detected during fungus growth in different lighting conditions.

Gene ID	Blast Best Hit	Signalling Pathway	Expression	
			Log2 Fold Change	*p*-Value
**White vs. Dark**				
XLOC_005484	Ras-related protein RABB1c	AMPK	1.747524	0.002175
XLOC_000988	Dihydroxyacetone kinase 2	RIG-I-like receptor	1.59843	7.42 × 10^−6^
XLOC_003649	Calcium-transporting ATPase sarcoplasmic/endoplasmic reticulum type	cGMP-PKG	1.344607	0.026421
XLOC_010509	Flocculation protein FLO11	MAPK	1.340754	0.009125
XLOC_002816	Transcription factor atf1	MAPK	1.325213	0.024773
XLOC_008849	Serine palmitoyltransferase 2	Increase in 3-dehydrosphinganine	1.145672	0.042199
XLOC_001876	Serine/threonine-protein phosphatase 2B catalytic subunit	cGMP-PKG/MAPK	1.068844	0.03373
XLOC_008348	Serine/threonine-protein kinase STK11	AMPK/FoxO	0.958811	0.008435
XLOC_002560	Mitogen-activated protein kinase dlk-1	MAPK	0.942856	0.00767
XLOC_010380	Receptor-interacting serine/threonine-protein kinase 1	RIG-I-like receptor	0.786022	0.015669
XLOC_000296	Protein phosphatase 2C homolog 1	MAPK	0.734535	0.010766
XLOC_003540	Striatin-3	MAPK	−0.66931	0.0101
XLOC_008420	DNA damage checkpoint protein rad24	MAPK	−0.79174	0.041947
XLOC_001763	ADP-ribosylation factor 6	Phospholipase D	−1.17631	0.003653
XLOC_000832	Sphingosine-1-phosphate lyase	Phosphoetanolamine decrease	−1.30105	0.018164
**Blue vs. Dark**				
XLOC_010509	Flocculation protein FLO11	MAPK	0.709652	0.026203
XLOC_009396	Catalase-1	MAPK/FoxO	0.590792	0.035802
**Red vs. Dark**				
XLOC_001068	Protein phosphatase PP2A regulatory subunit B	PP2A/AMPK	−0.79388	0.04389
